# Effects of Water Deuteration on Thermodynamic and Structural Properties of Proteins and Biomembranes

**DOI:** 10.1021/acs.jpcb.2c08270

**Published:** 2023-02-01

**Authors:** Carmelo Tempra, Victor Cruces Chamorro, Pavel Jungwirth

**Affiliations:** Institute of Organic Chemistry and Biochemistry of the Czech Academy of Sciences, 160 00 Prague 6, Czech Republic

## Abstract

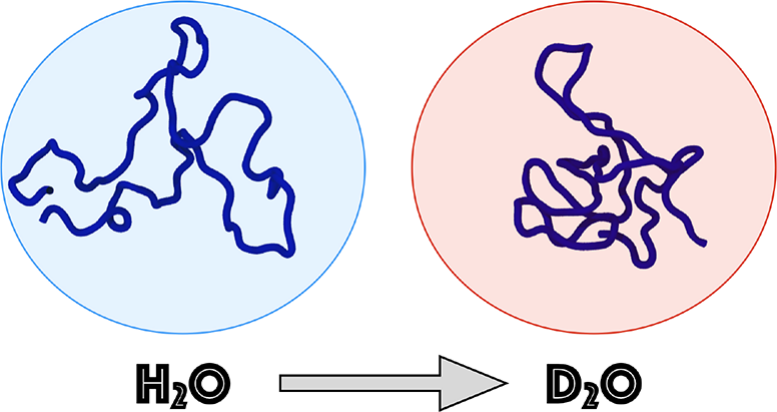

Light and heavy water are often used interchangeably in spectroscopic experiments with the tacit assumption that the structure of the investigated biomolecule does not depend too much on employing one or the other solvent. While this may often be a good approximation, we demonstrate here using molecular dynamics simulations incorporating nuclear quantum effects via modification of the interaction potential that there are small but significant differences. Namely, as quantified and discussed in the present study, both proteins and biomembranes tend to be slightly more compact and rigid in D_2_O than in H_2_O, which reflects the stronger hydrogen bonding in the former solvent.

## Introduction

Properties of light (H_2_O) and heavy (D_2_O) water are very similar to each other, save for a trivial ∼10% difference in density due to the higher mass of the D over the H isotope.^1^ This similarity is the rationale behind using the two water isotopes interchangeably as biomolecular solvents in spectroscopic experiments.^2,3^ Nevertheless, concerning nontrivial differences the two solvents vary by several degrees in melting points, by 0.4 pH (or pD) units in the autoionization equilibrium constant, and by about 20% in viscosity. Also, the number density (i.e., the number of molecules per unit volume) of D_2_O is not equal to, but is actually lower than, that of H_2_O. These variations in turn translate to a slightly different behavior of biomolecules dissolved in H_2_O vs in D_2_O. In particular, soluble proteins tend to be somewhat more compact and rigid in heavy water and so do phospholipid bilayers.^4,5^ As we have discussed recently, an intriguing consequence of nuclear quantum effects in water is the observed sweet taste of heavy water, as contrasted to a taste-neutral light water.^6^ More precisely, it is the reduction of nuclear quantum effects upon moving from H_2_O to D_2_O that triggers the activation of the human sweet taste receptor.

Small differences between light and heavy water can be related to slightly stronger hydrogen bonds in the latter liquid. These in turn can be traced back to nuclear quantum effects. Namely, zero point motions along as well as perpendicular to the direction of the water–water hydrogen bond are more pronounced in H_2_O over in D_2_O with a net effect of a slight hydrogen bond destabilization.^7^ Rigorously, computationally demanding quantum simulations such as path integral molecular dynamics (PIMD) should be employed to recover these effects. While feasible for neat water, such simulations become prohibitively expensive when large biomolecules are added to the solution. However, as already demonstrated by Feynmann and Hibbs, zero point energy effects can effectively be incorporated into classical simulations by modifying the interaction potential.^8,9^ We have recently employed this approach to develop, based on an earlier model,^10^ a classical force field for heavy water. Here, we use this approach to quantify the differences in thermodynamic and structural properties of amino acids, proteins, and phospholipid membranes in light vs heavy water, comparing the simulation result to experiment whenever possible and providing a molecular interpretation of the observed phenomena. Focusing on differences between bulk properties of light vs heavy water, secondary effects of deuteration of exchangeable hydrogens of the biomolecules in heavy water have been neglected in the present study.

## Methods

### Force Fields

The force fields used here to simulate H_2_O and D_2_O are the commonly used SPC/E model^11^ for the former and our recently developed SPCE-HW parametrization^10^ for the latter. Amino acids, proteins, lipids, and ions were modeled using the CHARMM36 topology generated by the CHARMM-GUI web interface.^12,13^ Classical equations of motion were solved numerically with a 2 fs integration time step using the Verlet-list algorithm.^14^ Long-range electrostatic interactions were accounted for using the Particle Mesh Ewald scheme^15,16^ employing a short-range cutoff of 1.2 nm. For the van der Waals interaction, a force-switching algorithm from 1.0 to 1.2 nm was employed. Simulations were run in the isothermal–isobaric (*NpT*) ensemble with the velocity-rescale thermostat^17^ and the Parrinello–Rahaman barostat^18^ imposing a temperature of 298 K and a pressure of 1 atm, with coupling constants of 5 and 1 ps, respectively.

### Free Energy of Amino Acid Transfer

**Figure 1:**
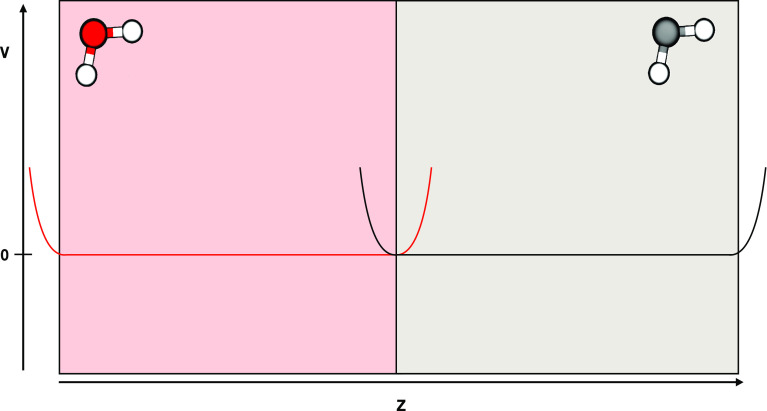
Simplified description of the setup used for calculating the free energy of transfer from H_2_O to D_2_O. Red (gray) color indicates D_2_O (H_2_O). The flat-bottomed potentials are also indicated in the figure.

A box 6 × 6 x 12 nm^3^ unit cell was filled with adjacent equally sized slabs of light (SPCE) and heavy (SPCE-HW) water, each containing 7203 water molecules. A flat-bottomed potential along the long (*z*) axis (see Figure 1) with a force constant of 100 kJ/mol and a distance from the center of each slabs *R*_*i*_ of 3 nm was applied to each of the cubes to keep the light and heavy water molecules separated from each other.

The system was energy minimized and then equilibrated in the *NpT* ensemble for 10 ns. All the essential amino acids were one by one placed in the center of the SPC/E water slab and energy minimized. The umbrella sampling technique was then applied to compute the Potential of Mean Force (PMF) along the *z* axis (moving from the H_2_O slab to D_2_O), and the free energy of transfer is extracted as the difference of the PMF at the bulk of the two solvents. Thirty windows were generated along the *z* axis, each separated by 0.2 nm, and a force constant of 1000 kJ mol^–1^ nm^–2^ was applied in each window. Free energies in the individual windows were connected using the weighted histogram analysis method (WHAM), with the associated statistical error evaluated using the bootstrap method.^19,20^

### Protein and Phospholipid Membrane Simulations

Three representative globular proteins have been chosen for the present study: azurine,^21^ lactoglobuline,^22^ and ribonuclease T1.^23^ The initial PDB structures were processed and solvated in a water box extending at least 2 nm from the protein to the edges of the unit cell using the CHARMM-GUI web server.^12,13^ The CHARMM-GUI default water model (i.e., TIP3P) was changed to SPC/E or SPCE-HW. Sodium or chloride counterions^13^ were added to neutralize the systems. The obtained systems were then energy minimized and equilibrated in the *NpT* ensemble for 10 ns, after which a production run of 1 μs followed for each of the three proteins. In addition, for ribonuclease a set of extra simulations in a range of different temperatures was performed in order to simulate the melting of the protein. For each temperature, a 1.7 μs trajectory was generated with the first μs taken as equilibration and discarded from the analysis (for further details see the Supporting Information, Tables S2 and S3).

To explore the effect of water deuteration on biological membranes, a bilayer containing 200 phospholipids (POPC) was constructed using CHARMM-GUI.^12,13^ The total amount of water molecules (SPC/E or SPCE-HW) added was 15180. After energy minimization and equilibration in the *NpT* ensemble of 10 ns, the systems were run for 200 ns. Furthermore, a patch of a dipalmitoylphosphatidylcholine (DPPC) membrane was built using CHARMM-GUI^12,13^ and simulated to evaluate the effect of the employed water models on the temperature of phase transition from the gel phase to the liquid phase. A bilayer composed of 64 lipids was solvated with a total of 2600 water molecules and simulated with temperature annealing from 325 to 305 K in 2 μs.

## Results and Discussion

**Figure 2:**
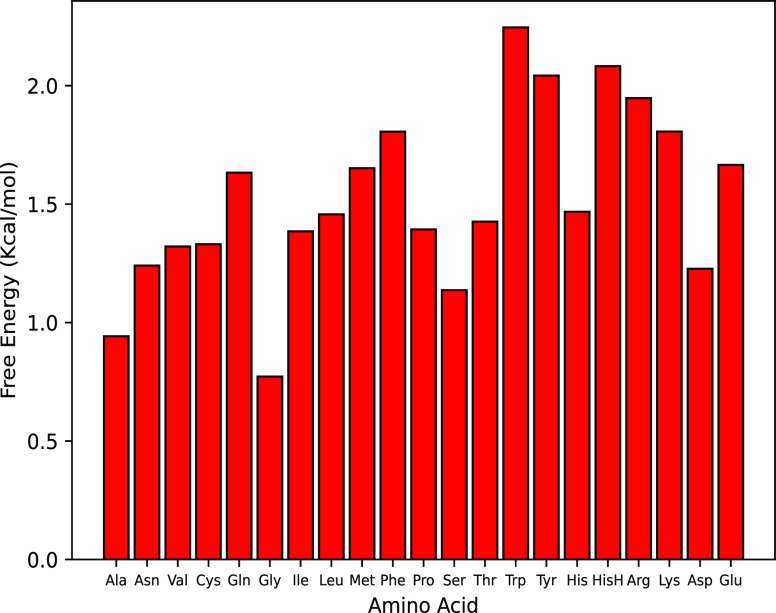
Free energy of transfer from SPC/E (H_2_O) to SPCE-HW (D_2_O). Errors are not reported because too small. A table of the values with associated errors can be found in the Supporting Information (Table S1).

**Figure 3:**
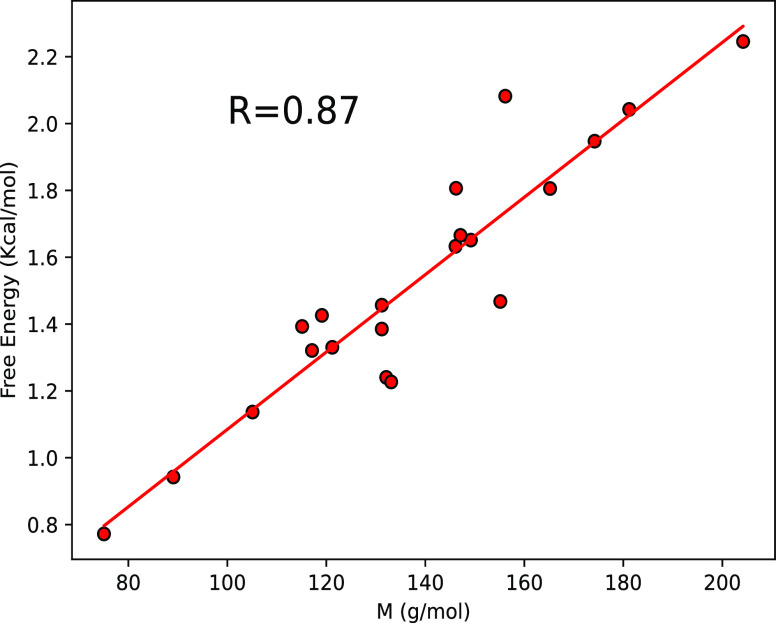
Free energy of transfer from SPC/E (H_2_O) to SPCE-HW (D_2_O) as a function of the amino acid molar mass showing a very good linear correlation.

The free energies of transfer Δ*G* from H_2_O to D_2_O are summarized for all the amino acids in Figure 2. All the calculated free energies are positive, which means the amino acids are less stable in the heavy water than in light water. It is worth noting that the Δ*G* values, which vary between 0.7 and 2.2 kcal/mol do not follow the hydrophobicity scale of amino acids. The results presented in Figure 2 rather point to the molecular size as the main factor governing the Δ*G* values: the larger the amino acid, the more unfavorable the transfer from H_2_O to D_2_O. The always positive free energy of transfer between the two solvents indicates that, compared to H_2_O, D_2_O has a higher propensity to form water–water hydrogen bonds than water–amino acid hydrogen bonds. This explains why the free energy of hydration depends on the excluded volume, which for small molecules like amino acids correlates well with the molecular weight. We indeed see a very good linear correlation between the molar mass and the free energy of transfer (Figure 3).

**Figure 4:**
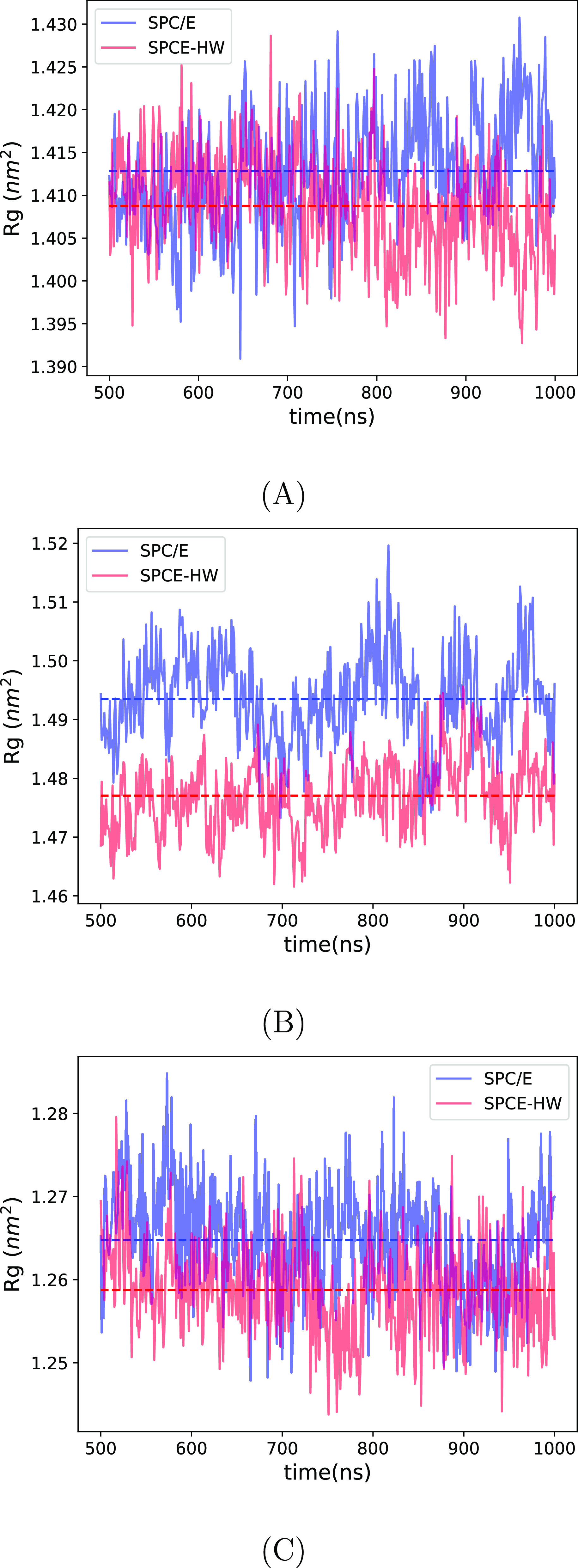
Radius of gyration of azurine (A), lactoglobuline (B), and ribonuclease (C) in SPC/E (H_2_O, blue line) and SPCE-HW (D_2_O, red line). The dashed lines represent average values over the production runs of 500 ns.

The above results concerning amino acids indicate that D_2_O may be a somewhat worse solvent than H_2_O for proteins, hence inducing also more compact structures with a reduced radius of gyration. To test this, we modeled and analyzed the behavior of three globular proteins (azurine, lactoglobuline, and ribonuclease) in D_2_O vs H_2_O. All three proteins show a small but consistent decrease in the radius of gyration when moving from H_2_O to D_2_O; see Figure 4. The same trend is also observed for the solvent accessible surface area (SASA); see Figure S1 in the Supporting Information. Simulations thus show that water deuteration is making the proteins tighter, which is consistent with the positive free energy of transfer presented above, as well as with the generally tightening effect of D_2_O found in experiment.^24^

**Figure 5:**
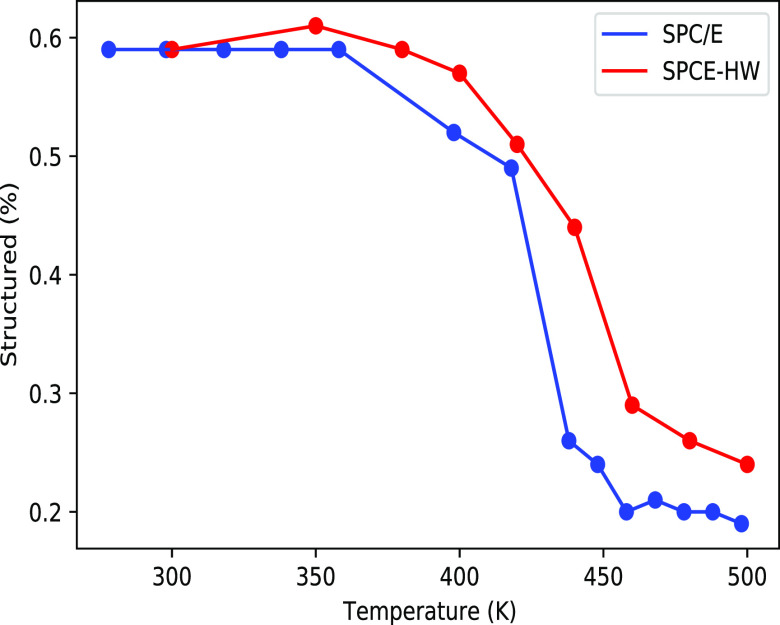
Annealing of ribonuclease. The percentage of structured protein is plotted versus the temperature. The percentage of structured protein was calculator using the gromacs tool “gmx do dssp”, which uses the DSSP algorithm.^25,26^ The blue line is SPC/E (H_2_O), and the red line is SPCE-HW (D_2_O). Error bars are not reported as they are too small to be visible in the figure (i.e., below 1%).

For ribonuclease, we also modeled the effect of deuteration on the protein melting temperature. From the results presented in Figure 5, we see that the tightening of the protein structure upon water deuteration also leads to stabilization and increase of the protein melting temperature.

This is in line with experimental observations,^27,28^ and it is consistent with the sign of the free energies of transfer from H_2_O to D_2_O of individual amino acids in Figure 2.

The tightening effect of the heavy water is not limited to proteins as demonstrated on the areas per lipid (APL) calculated for a POPC bilayer, yielding a value of 0.63 nm for H_2_O and 0.59 nm for D_2_O. The effect of the solvent on the POPC membrane APL is qualitatively in line (albeit more pronounced) with previous simulations.^29^ At the same time, we observe a small (about 3%) bilayer thickening, as deduced from the density profile of phosphate (see the Supporting Information). These results are consistent with experimental findings.^30^

**Figure 6:**
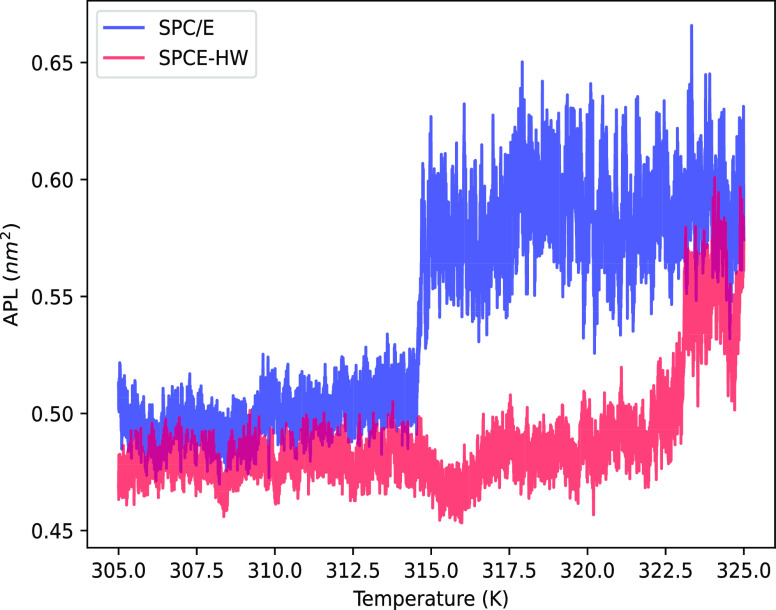
Area per lipid of a DPPC bilayer as a function of temperature in SPC/E H_2_O (blue) and SPCE-HW D_2_O (red).

In addition, the behavior of a DPPC bilayer around the melting point was investigated by monitoring the APL as a function of temperature. The results are shown in Figure 6. The first thing to notice is that the APL in D_2_O is systematically smaller than that in H_2_O, which is consistent with the above results for POPC. DPPC in H_2_O exhibits a melting temperature of 314.5 K, which is in very good agreement with both the experimentally determined value of 314.15 K^31^ and the value from MD simulations using the default TIP3P charmm model.^32^ The system simulated in D_2_O using the present model shows a clear upward shift of the melting temperature of almost 10 K. Qualitatively, this is in accord with experimental findings,^31^ although the measured shift in melting temperature is smaller (less than 1 K).

## Conclusion

In this work, we quantified the effect of water deuteration on amino acids, proteins, and phospholipid membranes. This was done using classical molecular dynamics simulations employing models that account for differences between H_2_O and D_2_O in an effective way, incorporating nuclear quantum effects into the intermolecular potential. In particular, we focused on differences in structural properties such as the compactness of the biomolecules and thermodynamic effects like melting temperatures and free energies of transfer of solutes from light to heavy water. To the former, our results reveal small but systematic structural effects on proteins. Namely, we observe a decrease in radii of gyration of less than 1% upon moving from H_2_O to D_2_O. Interestingly, structural effects on phospholipid membranes are larger than on proteins; in particular, upon deuteration we observed a decrease of the area per lipid by more than 10% and thickening of the bilayer by about 3%. To the latter, our results show that all amino acids are slightly less soluble in heavy vs light water. Also, moving from H_2_O to D_2_O, we observe an upward shift by several degrees of the melting point of a model protein (ribonuclease). The same affect of increasing the melting temperature is found for DPPC. Altogether the simulations show that the structural effect on a globular protein might be small, but the thermodynamic effect (melting) on protein and membranes can be important, especially if an experiment is conducted close to the phase transition temperature, where even a small shift can change the physical-chemical properties. Comparison to available experimental data shows that our simple models capture well the principal effect, namely, that D_2_O is a somewhat worse solvent for biomolecules that H_2_O. This also implies that association between proteins or between a protein and a biomembrane may be positively affected by water deuteration. Finally, protein domains that are intrinsically disordered and thus very sensitive to the balance between protein–solvent and solvent–solvent interaction, may show a high sensitivity to the H_2_O to D_2_O substitution.

## Supporting Information Available

Free energy of transfer (with associated error) between water and heavy water for each single amino acid, solvent accessible surface area (SASA) of azurine, lactoglobulin, and ribonuclease in water and heavy water. Detailed data about annealing of ibonuclease T1. Phosphate density profile of POPC in water and heavy water (PDF)
