# Supplementary material for: Effects of Water Deuteration on Thermodynamic and Structural Properties of Proteins and Biomembranes
Source: J Phys Chem B. 2023 Feb 1;127(5):1138–43. doi: 10.1021/acs.jpcb.2c08270 (PMC10017026; doi:10.1021/acs.jpcb.2c08270)
Supplement: Supplementary file 1 — jp2c08270_si_001.pdf [file jp2c08270_si_001.pdf]

Supporting Information: Effects of Water Deuteration on Thermodynamic and Structural Properties of Proteins and Biomembranes

Carmelo Tempra, Victor Cruces Chamorro, and Pavel Jungwirth*

Institute of Organic Chemistry and Biochemistry of the Czech Academy of Sciences, Flemingovo nám. 542/2, 160 00 Prague 6, Czech Republic

E-mail: pavel.jungwirth@uochb.cas.cz

Free energy of transfer from H_2O to D_2O

aa	$\Delta G(Kcal/mol)$	Error $(Kcal/mol)$
Ala	0.942	0.011
Asn	1.240	0.010
Val	1.320	0.009
Cys	1.330	0.018
Gln	1.632	0.010
Gly	0.771	0.018
Ile	1.385	0.012
Leu	1.456	0.011
Met	1.651	0.015
Phe	1.805	0.012
Pro	1.392	0.013
Ser	1.136	0.010
Thr	1.425	0.014
Trp	2.245	0.014
Tyr	2.042	0.014
His	1.464	0.011
HisH	2.082	0.012
Arg	1.947	0.009
Lys	1.806	0.010
Asp	1.226	0.009
Glu	1.665	0.013

Table S1: Free energy of transfer of each single amino acid from H_2O to D_2O, calculated using PMF and WHAM.

Solvent Accessible Surface Area (SASA)

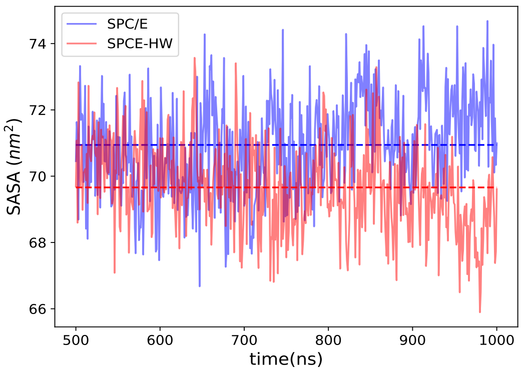

(A)

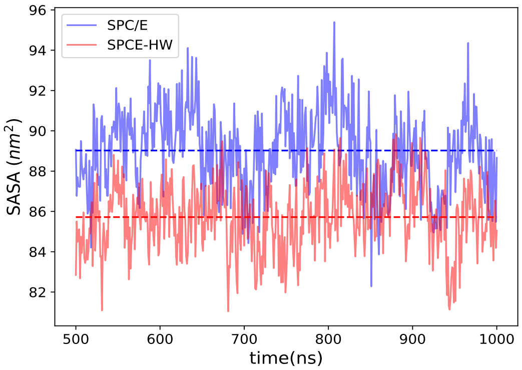

(B)

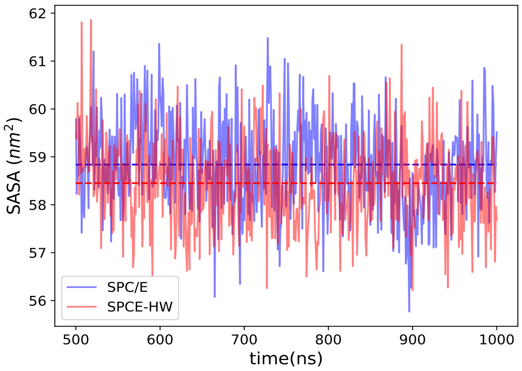

(C)

Figure S1: SASA of azurine (A), lactoglobuline (B) and ribonuclease (C) in SPC/E (H_2O, blue line) and SPCE-HW (D_2O, red line). The dashed lines represent average values over the production runs of 500 ns.

Annealing of ribonuclease T1

T(K)	% Structured
278	0.59
298	0.59
318	0.59
338	0.59
358	0.59
398	0.52
418	0.49
438	0.26
448	0.24
458	0.20
468	0.21
478	0.20
488	0.20
498	0.19

Table S2: Pecentage of structured protein (calculated with DSSP algorithm), for ribonuclease T1 in H_2O

T(K)	% Structured
300	0.59
350	0.61
380	0.59
400	0.57
420	0.51
440	0.44
460	0.29
480	0.26
500	0.24

Table S3: Pecentage of structured protein (calculated with DSSP algorithm), for ribonuclease T1 in D_2O

POPC membrane

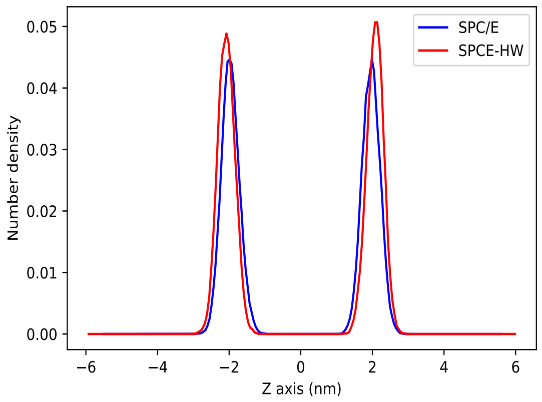

Figure S2: Phosphate density profile in SPC/E (H_2O, blue line) and SPCE-HW (D_2O, red line).
